# *Catsper1* promoter is bidirectional and regulates the expression of a novel lncRNA

**DOI:** 10.1038/s41598-017-13867-2

**Published:** 2017-10-17

**Authors:** Salma E. Jiménez-Badillo, Norma Oviedo, Christian Hernández-Guzmán, Lorenza González-Mariscal, Javier Hernández-Sánchez

**Affiliations:** 1 Departamento de Genética y Biología Molecular, Centro de Investigación y de Estudios Avanzados del IPN, Ciudad de México, Mexico; 2 Unidad de Investigación Médica en Inmunología e Infectología, Centro Médico Nacional, La Raza, Instituto Mexicano del Seguro Social (IMSS), Ciudad de México, Mexico; 3 Departamento de Fisiología, Biofísica y Neurociencias, Centro de Investigación y de Estudios Avanzados del IPN, Ciudad de México, Mexico

## Abstract

The *Catsper1* gene, whose expression is restricted to male germ cells, has great importance in reproductive biology because of its function in sperm motility and fertilization. We previously reported that the promoter of this gene has transcriptional activity in either direction in a heterologous system. In the present study, we found that the *Catsper1* promoter has *in vitro* transcriptional activity in either orientation in GC-1 spg mouse spermatogonial cells. The results also showed that this promoter regulates the expression of a new divergent *Catsper1* gene named *Catsper1au* (*Catsper1* antisense upstream transcript). *Catsper1au* is expressed in adult male mouse testis and liver tissues but not in female mouse liver or ovary tissues. In the testis, *Catsper1au* is expressed in embryos at 11.5 days post-coitum and from newborns to adults. This gene is also expressed in 1- to 3-week postnatal hearts and in 1-week to adult stage livers. The analysis of the 1402 bp whole genome sequence revealed that *Catsper1au* is an intronless and polyadenylated lncRNA, located in the nuclei of Sertoli and spermatogenic cells from adult testis. These data indicate that *Catsper1au* is divergently expressed from the *Catsper1* promoter and could regulate gene expression during spermatogenesis.

## Introduction

The genes expressed during spermatogenesis encode proteins and non-coding RNAs, which enable the progress of specific processes necessary for germ cell development^1,2^. The activation of these genes is highly controlled and includes mechanisms of transcriptional regulation in *cis* or *trans* that involve promoters, alternative splicing, transcriptional factors, enhancers, epigenetic mechanisms, and the more recently studied noncoding RNAs^3–5^. Male germ cells produce transcripts homologous to genes expressed in somatic cells, such as *Gapdhs* and alternative transcripts (*Hk1s*) expressed from the same gene in somatic cells^6^. There are also unique transcripts that do not have similarity to any other somatic transcripts, such as *Prm1* and *Catsper* ^3,7^, and transcripts that encode proteins and non-coding RNAs whose function has not yet been characterized. CATSPER is a cation channel exclusively detected in sperm cells and located in the plasma membrane of the principal piece of the flagellum. CATSPER comprises a 4-protein family forming a tetrameric channel (CATSPER 1–4)^8–10^. The 4-member family associates with accessory proteins (CATSPERβ, CATSPERγ, and CATSPERδ)^11–13^. This channel mediates Ca2+ entry necessary for the hyperactivation motility and fertility since it is required to penetrate the zona pellucida surrounding the oocyte^7^. The four subunits are expressed at different stages of spermatogenesis. While *Catsper1*, *3* and 4 are transcribed in spermatids, indicating common transcriptional regulation, *Catsper* *2* is found in pachytene spermatocytes^7,14–16^. The *Catsper1* gene was the first discovered in this family and differs from the other familial genes in its histidine-rich cytoplasmic domain, which senses changes in intracellular pH and activates the CATSPER channel^7,17^. However, the disruption of any of the four *Catsper* genes results in a nonfunctional channel leading to deficient hyperactivation motility and male infertility^16^. However, mutations in *CATSPER1* and *2* have been associated with human infertility^18,19^. These facts make the CATSPER channel a target for developing a male contraceptive and for the study of male infertility. Previously, we characterized the promoter of human and mouse *Catsper1* genes to understand the molecular mechanisms underlying the transcriptional regulation of this gene. Although in a heterologous system, the mouse *Catsper1* promoter showed unusual bidirectional transcriptional activity even higher in antisense than in sense (*Catsper1*) orientation^20^. These data suggest that the mouse *Catsper1* promoter could be the bidirectional promoter responsible for the transcription of a new gene in the antisense strand. However, there are no annotated genes in the antisense orientation in the 10.4-kb region upstream of *Catsper1* transcriptional start site (TSS) and downstream of the *Gm7074* pseudogene. Therefore, in the present study, the bidirectional transcriptional activity of the *Catsper1* promoter was analysed in a homologous system and whether a new divergent *Catsper1* gene is expressed. The *Catsper1* promoter gene showed bidirectional transcriptional activity in spermatogonial GC-1 spg cells. Two TSSs were determined using 5′-RACE for the new gene named *Catsper1au* (*Catsper1* antisense upstream transcript) in adult testis. Unlike *Catsper1*, the antisense transcript was not exclusive of the testis. The *in silico* analysis of the whole sequence revealed that *Catsper1au* is polyadenylated, intronless and does not contain translatable open reading frames (ORF). No protein was produced in an *in vitro*-coupled transcription and translation assay. *Catsper1au* was restricted to the nucleus of spermatogenic and Sertoli cells.

## Results

**Figure 1:**
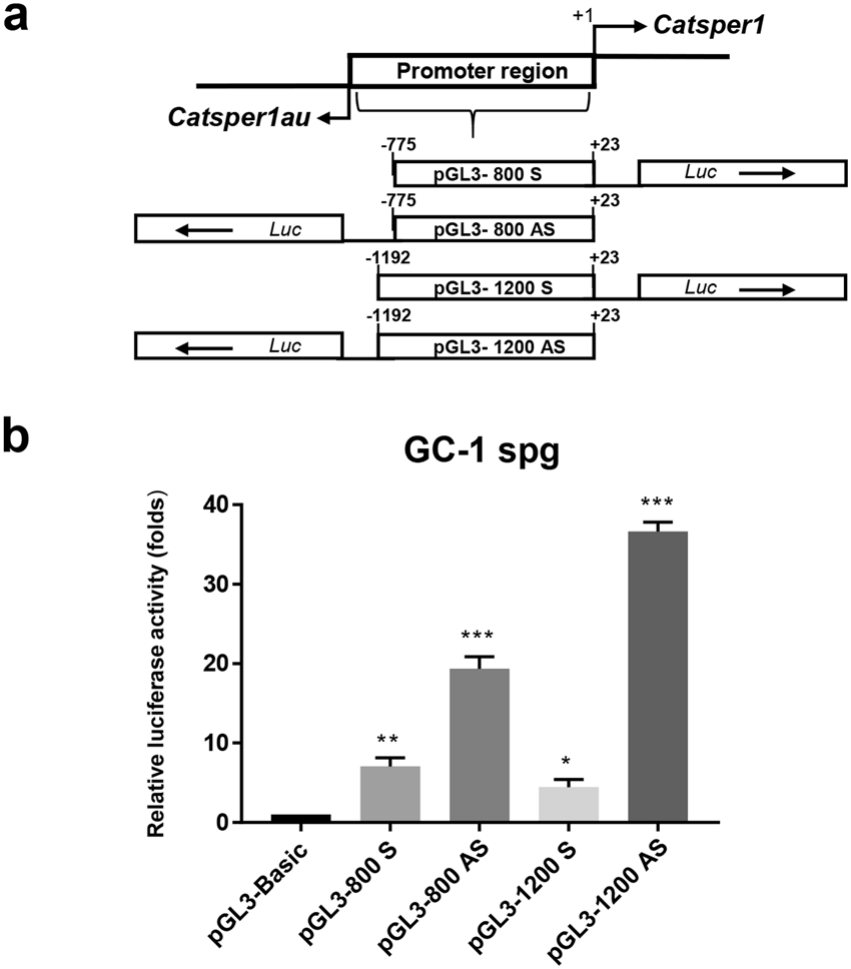
The *Catsper1* promoter has bidirectional transcriptional activity in GC-1 spg spermatogonia cells. (**a**) Structure of the *Catsper1* promoter constructs. Each construct was named according to the length of the promoter region inserted in either direction upstream of the *Photinus* luciferase gene (*Luc*). (**b**) Transcriptional activity in GC-1 spg spermatogonia cells. Transcriptional activity is expressed as a fold-increase of luciferase activity over the pGL3-Basic (empty vector), to which a value of 1 was assigned. The results represent the average activity of *Photinus* firefly luciferase normalized to the activity of *Renilla* reniformis luciferase as an internal transfection control. Bar graphs represent the means ± SD, n = 3. Statistical significance was evaluated with one-way ANOVA: *P < 0.05, **P < 0.01, ***P ≤ 0.001.

*Catsper1* promoter has antisense transcriptional activity in mouse spermatogonial GC-1 spg cells. Previous results have suggested that the *Catsper1* promoter may act as a bidirectional promoter driving the expression of a divergent gene to *Catsper1* ^20^. To determine whether this promoter presents bidirectional transcriptional activity in a homologous system, spermatogonial (GC-1 spg) cells were transiently transfected with pGL3-800 and pGL3-1200 constructs. These constructs contain the *Catsper1* promoter regions in either direction (coordinates −75 to +23 and −1192 to +23, relative to *Catsper1* TSS + 1) upstream of the *Photinus* luciferase reporter gene (Fig. 1a). Both the 800- and 1200-bp promoter regions showed bidirectional activity; however, their antisense (*Catsper1au*) activities were up to 19.3- and 36.6-fold higher, respectively, while sense (*Catsper1*) activities showed only 7- and 4.4-fold higher activity compared with pGL3-Basic vector (Fig. 1b). These results indicate that the *Catsper1* promoter has bidirectional transcriptional activity in spermatogonial cells.

### *In silico* analysis of the 10.4-kb region upstream of the mouse *Catsper1* gene: Identification of the TSS of the new *Catsper1au* gene

**Figure 2:**
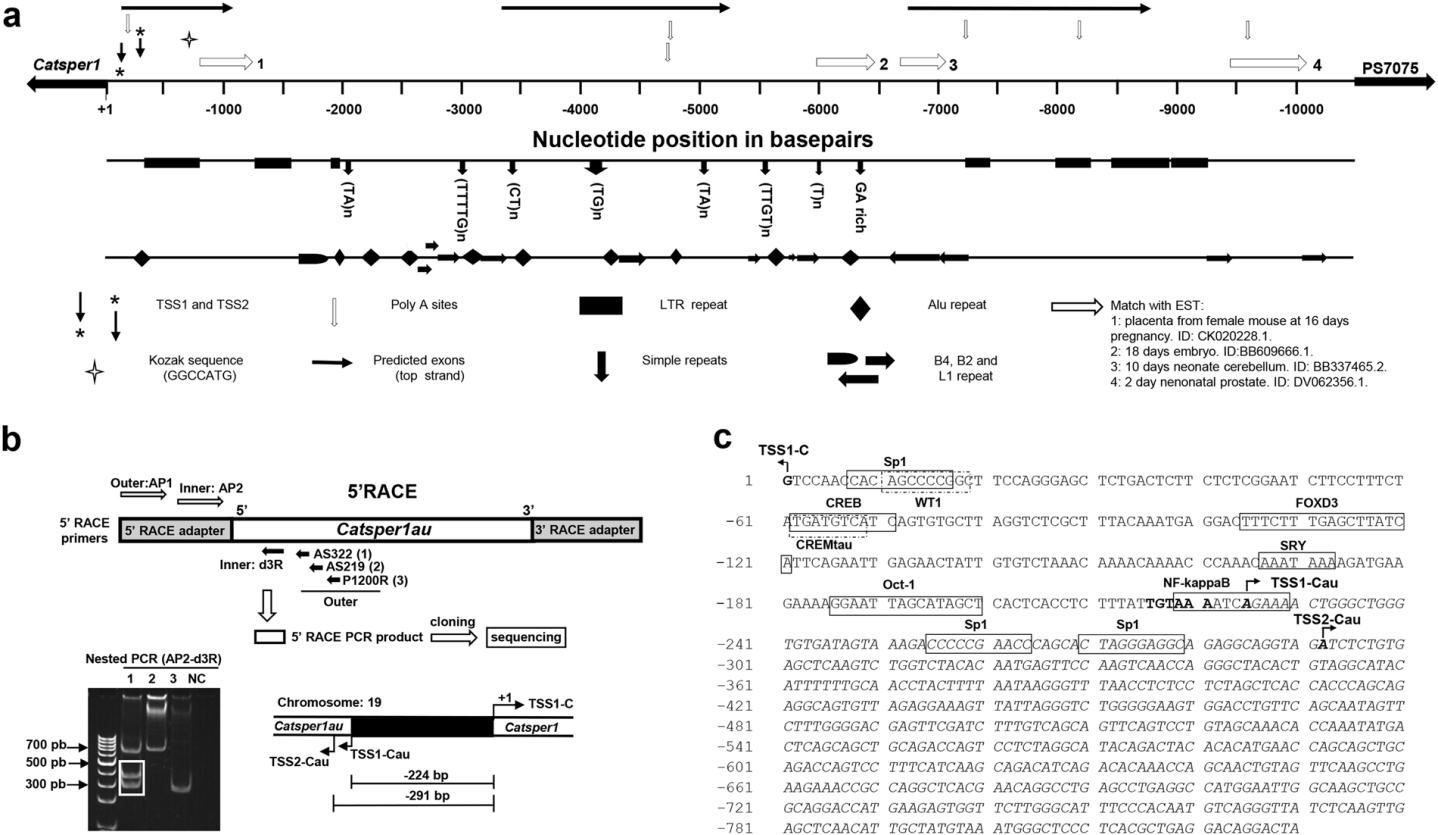
*In silico* analysis of the 10.4 kb region upstream of the mouse *Catsper1* gene and determination of the divergent gene TSSs. (**a**) The new putative *Catsper1* divergent gene and its structure were predicted using different web servers (see Material and Methods). *Catsper1* TSS is indicated. The new gene putative TSSs predicted at −121 and −289 bp are indicated. Predicted poly(A) and Kozak sequences, as well as exons and matches to EST 1–4, are indicated. LTR, simple repeats, Alu, B4, B2, and L1 repeat sequences are also indicated. (**b**) TSSs of the new gene *Catsper1au*. TSS were determined by 5′-RACE using a nested PCR as outlined in Materials and Methods. Two *Catsper1au* TSSs were confirmed as indicated by the sequencing of the 300 and 350 bp bands obtained in the second reaction of the nested PCR with primers AP1-AS322 (boxed in lane 1). The distances between *Catsper1au* TSS1 and TSS2 (labelled as TSS1-Cau and TSS2-Cau) from *Catsper1* TSS-C were 224 and 291 bp, respectively. (**c**) Schematic representation of the *Catsper1* bidirectional promoter in testis. Bent arrows indicate TSS-C (+1 bp) and TSS1-Cau (−225) and TSS2-Cau (−292). The arrows represent the direction of gene transcription. The predicted cis-acting elements in the 5′ upstream region of *Catsper1au* (transcriptional factors) are boxed; the TATA box (−216 bp) is bolded. The 5′ *Catsper1au* sequence, determined using 5′-RACE, is shown in italics.

According to the mouse genome NCBI database, there are no annotated genes in the region upstream of *Catsper1* in the complementary DNA chain. However, the antisense transcriptional activity of the *Catsper1* promoter *in vitro* suggests the presence of a divergent gene in this region. Therefore, an *in silico* analysis of the 10.4-kb region downstream of the *Gm7074* pseudogene (ID: 631868) and upstream of *Catsper1* gene (ID: 225865) was performed (Fig. 2a). The sequence showed a Kozak sequence, poly (A) sites, and two TSSs, at 121 and 289 bp upstream of *Catsper1* TSS, but no CpG islands were detected. Several matches with EST databases were also found. The analysis of this region also indicated a GC content of 45.10%. The presence of splicing donor/acceptor sites in this region predicted a putative *Catsper1* divergent gene encoding a 4.4-kb transcript with three exons. Repetitive sequences are primarily located in the non-transcribed genomic regions or within intronic sequences^21,22^. The *in silico* analysis revealed that 58.02% of the 10.4 kb region contains repeats, such as: long terminal repeats (LTR); short interspersed nuclear elements (SINE), which include Alu (B1), B2 and B4; and long interspersed nuclear elements (LINE), such as L1 and simple repeats, whose content was 19.21, 28.23, 5.91 and 4.18% (Fig. 2a).

Bidirectional promoters regulate the transcription of genes expressed in the same tissue and frequently have related functions^23,24^. Since *Catsper1* is expressed only in spermatogenic cells^7^, we performed a 5′-RACE from adult mouse testis RNA to analyse whether the transcript predicted *in silico* was indeed expressed and to determine the distance between the new gene and *Catsper1* TSS. To this end, primers specific for sequences within the first predicted exon were tested in nested PCR (Table 1 and see Materials and Methods). The amplified fragments from three different reactions were cloned into pJET and subjected to sequence analysis to confirm their identity (Fig. 2b). Two TSSs were identified for the new gene *Catsper1au* at −224 and −291 bp from the *Catsper1* TSS (labelled as TSS1-C). The other products were nonspecific amplifications. Thus, the shortest intergenic distance was less than 1 Kb. These experimental TSSs were near the TSSs predicted *in silico* using the Network promoter programme. The intergenic sequence between *Catsper1* and *Catsper1au* showed a TATA box located at −216 bp relative to TSS-C, and the presence of putative testis-specific transcription factor-binding sites, such as CREMτ and SRY^25,26^ (Fig. 2c).

**Table 1:** Primers used.

Name	Sequence (5′→3′)	Size	Experiment
d3R (R)	AGACCCTAATAACTTTCCTCTAACACTGCCTCTGC	35	5′-RACE
AS322 (R)	TAGCTCCCTCTTAGTCCTGTC	21	5′-RACE
AS219 (R)	CCAGTTGAGTGATGACGGTC	20	5′-RACE
P1200 (R)	AGGGGTAACTTGGAGGAT	18	5′-RACE
Adaptor Primer 1(AP1) (F/R)	CCATCCTAATACGACTCACTATAGGGC	27	5′/3′-RACE
Nested Adaptor Primer (AP2) (F/R)	ACTCACTATAGGGCTCGAGCGGC	23	5′/3′-RACE
Act (F)	TGACGGGGTCACCCACACTGTGCCCATCTA	30	RT-PCR
Act (R)	CTAGAAGCATTTGCGGTGGACGATGGAGGG	30	RT-PCR
Cat1 (F)	CTGAGCTAGAGATCCGAGGTG	21	RT-PCR
Cat1 (R)	CAATTAGCTTGAGGACTGCTTCT	23	RT-PCR
NGFORW225 (F)	AGAAAACTGGGCTGGGTGTGATAGTAAAG	29	RT-PCR, *Catsper1au* Cloning
NGFORW292 (F)	ATCTCTGTGAGCTCAAGTCTGGTCTACAC	29	RT-PCR
AS306REV (R)	GGCAGCTTGCCAATTCCATGGCCTCAGG	28	RT-PCR
NGFORW225RT(F)	AGAAAACTGGGCTGGGTGTGATAG	24	qPCR
REVD3(R)	TGGTGAGCTAGAGGAGAGGTTAAAC	25	qPCR
ActBForw (F)	AAGATCAAGATCATTGCTCCTCC	23	qPCR
ActBRev (R)	TAACAGTCCGCCTAGAAGCA	20	qPCR
3′RACE-Forw1 (F)	CAGAGGCAGTGTTAGAGGAAAGTTATTAG	31	3′RACE
3′RACE-Forw2 (F)	GCTGAGGACAGGACTAAGAGGGAGC	25	3′-RACE
NG-Reverse (F)	CGGACTTGATTTGGCATTACCCTAATGGG	29	RT-PCR, *Catsper1au* Cloning
ENGFORW225 (F)	GAATTCAGAAAACTGGGCTGGGTGTGATAGTAAAG	35	*Catsper1au* Cloning
BRACE3REV (R)	GGATCCTCGGACTTGATTTGGCATTACCCTAATGGG	36	*Catsper1au* Cloning
Rnu1a1 (F)	ATACTTACCTGGCAGGGGAGA	21	RT-PCR
Rnu1a1 (R)	CAGGGGAGAGCGCGAACGCA	20	RT-PCR
HNGF225 (F)	AAGCTTAGAAAACTGGGCTGGGTGTGATAGTAAAG	35	RNA-FISH/NB
ECOAS306 (R)	GAATTCGGCAGCTTGCCAATTCCATGGCCTCAGG	34	RNA-FISH/NB
ActH3 (F)	AAGCTTTGACGGGGTCACCCACACTGTGCCCATCTA	36	RNA-FISH
ActECO (R)	GAATTCCTAGAAGCATTTGCGGTGGACGATGGAGGG	36	RNA-FISH

Abbreviations: R, reverse; F, forward; NB, Northern blot.

### Expression profile of *Catsper1au* in different tissues

**Figure 3:**
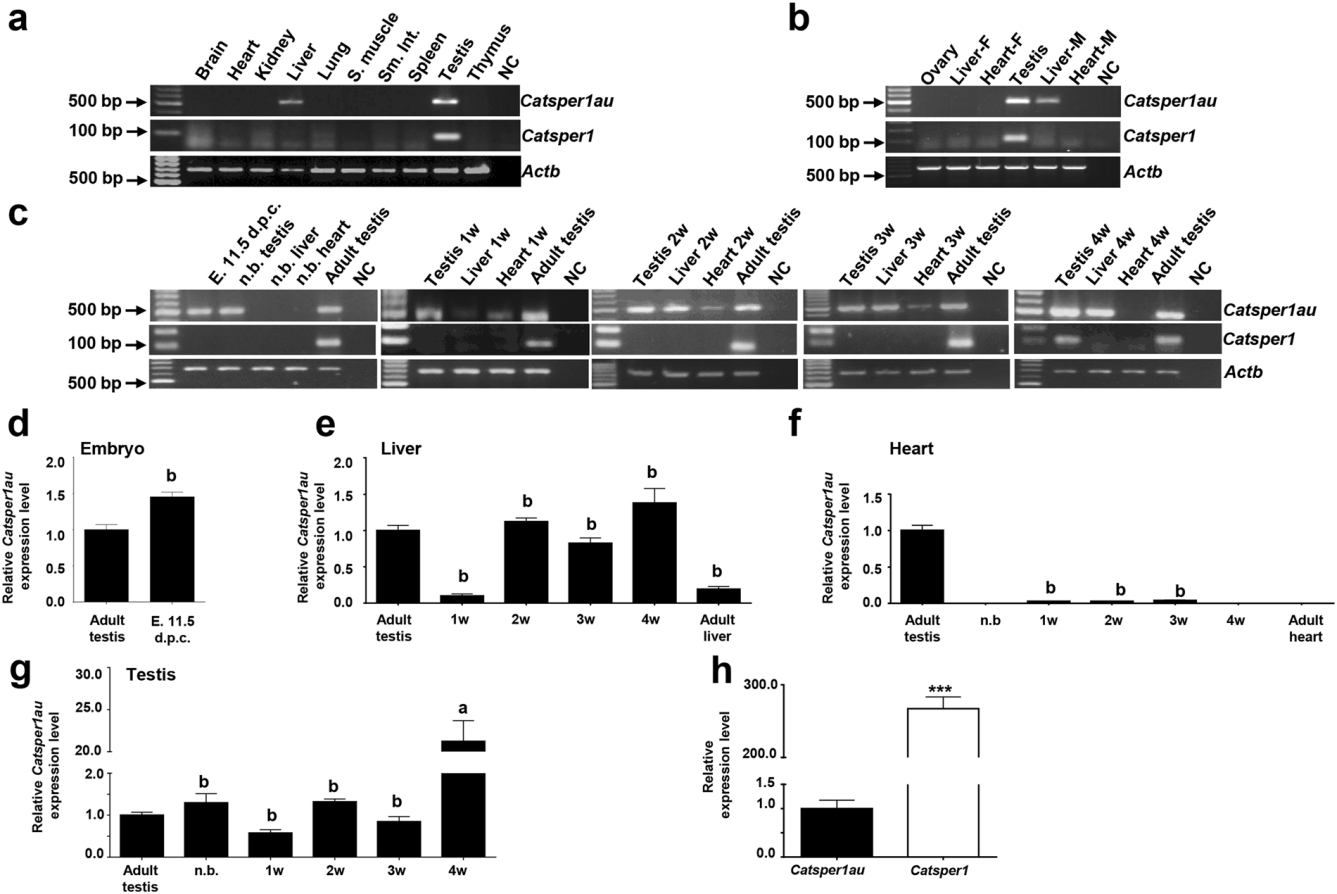
Expression profile of *Catsper1au* in male and female mouse organs. A cDNA panel was prepared from different organs and cells to amplify the *Catsper1au* 5′ region through PCR based on the sequences obtained using 5′-RACE. A product of 496 bp was produced using oligonucleotides AS-306REV and NG-FORW292. (**a**) *Catsper1au* expression from different adult mouse organs. S: smooth; Sm. Int.: small intestine. (**b**) *Catsper1au* expression in adult male and female gonads and organs. F: Female. M: Male. (**c**) *Catsper1au* expression profile at different stages of male mouse development. E: embryo; d.p.c: days post-coitum; n.b: newborn. W: week. A product of 93 bp was amplified for *Catsper1* in 4-week and adult testis. *Actb* was analysed as an internal expression control and to verify the absence of genomic DNA. The negative control (NC) is shown in the last lane. (**d**–**g**) *Catsper1au* RNA expression levels analysed using qRT-PCR in different tissues. Expression levels were normalized with respect to *Actb* mRNA. Values are expressed as fold-variations of each tissue type relative to adult testis (to which a value of 1 was assigned). Statistical significance was evaluated using one-way ANOVA: a > b. (**h**) *Catsper1* expression relative to *Catsper1au*. *Catsper1* was expressed as fold-changes in the expression of *Catsper1au* (***P < 0.001; paired t test). The results are expressed as the means ± S.E.M., n = 3 for each tissue.

To ascertain whether similar to *Catsper1*, Catsper1au is only expressed in adult mouse testis, RT-PCR from various adult tissues was performed using oligonucleotides based on the 5′-mRNA sequence obtained by 5′-RACE. As a control, *Actb* expression was analysed. Surprisingly, *Catsper1au* was also expressed in the liver (Fig. 3a) but not in the adult brain, heart, kidney, lung, skeletal muscle, small intestine, spleen, or thymus. Additionally, *Catsper1au* was only detected in adult male mice since no signal was observed in adult female ovary, liver, or heart tissues (Fig. 3b). The expression profile of *Catsper1au* was also analysed at different stages during male mouse development. *Catsper1au* was expressed at the embryonic stage (11.5 d.p.c.), newborn testis and continued until the adult stage. The expression was detected from the first week until the adult stage in the liver. Interestingly, this gene was also expressed in heart tissue from 1-to 3-week-old mice (Fig. 3c). To verify these results and assess the relative expression levels of *Catsper1au* RNA, qRT-PCR was performed in tissues where *Catsper1au* was detected using RT-PCR and tissues where *Catsper1au* was not amplified, including adult, new born and 4-week heart (Fig. 3d–g). Interestingly, *Catsper1au* RNA expression was significantly higher in 4-week testis compared with adult testis (Fig. 3d). This finding may be relevant because this lncRNA could play an active role in adolescent mice, where *Catsper1* expression is initiated in CD-1 mice (Fig. 3c). However, *Catsper1* mRNA expression in adult mouse testis was higher compared with *Catsper1au* RNA expression (Fig. 3e).

### *Catsper1au* is a lncRNA

**Figure 4:**
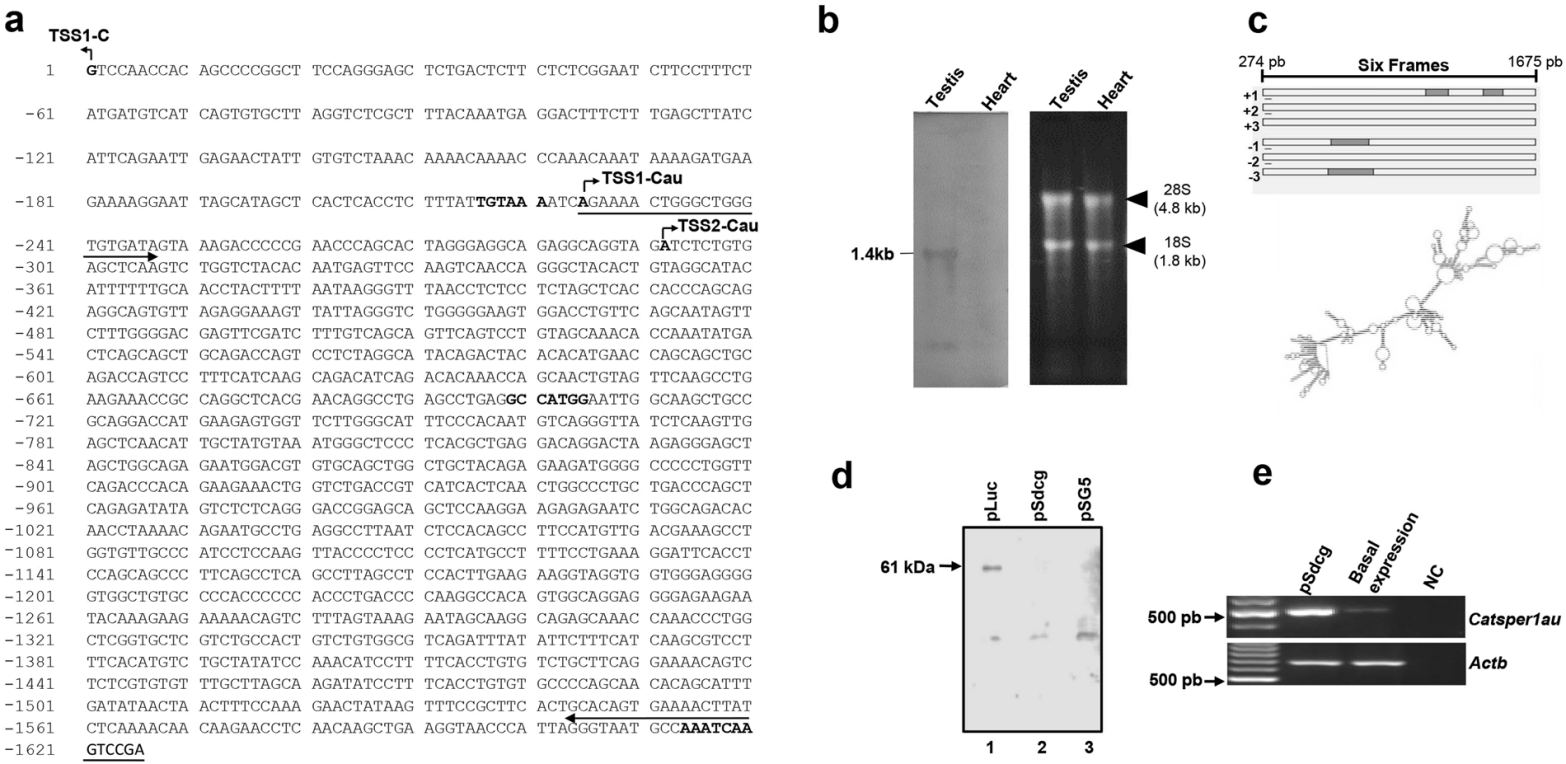
Sequence and characteristics of *Catsper1au*. The full length of the *Catsper1au* transcript (1402 bp) was determined by 5′/3′-RACE, cDNA cloning into pCG5 vector and sequencing. (**a**) A Kozak sequence (−699 bp) and the poly (A) signal sequence (−1,614 bp) are bolded. Bent arrows indicate TSS-C (+1 bp), TSS1-Cau (−225) and TSS2-Cau (−292). The 1402 bp transcribed region is indicated by the two bolded arrows from −225 to −1626 bp. (**b**) Expression of the 1.4 kb *Catsper1au* RNA in mouse adult testis. Total RNA from adult testis and heart was examined by Northern blot analysis using a digoxigenin-UTP-labelled riboprobe for *Catsper1au* (left panel). Ethidium bromide staining of ribosomal RNA (right panel). (**c**) The *in silico* analysis in all of the six reading frames did not reveal long open reading frame (ORF). (**c**) Predicted secondary structure of *Catsper1au* transcript. (**d**) *Catsper1au* transcript is not translated. A rabbit reticulocyte lysate system was used to express *Catsper1au* from pSG5 *in vitro*. Positive control, a construct coding 61-kDa luciferase (lane 1); pSdC1, a pSG5 derivative containing the 1.4 Kb RNA gene downstream of T7 promoter (lane 2); negative control, empty vector (pSG5) (lane 3). (**e**) Overexpression of *Catsper1au* RNA by RT-PCR in mouse germ cells. *Actb* was analysed as an internal expression control to verify the absence of genomic DNA.

*Catsper1au* was characterized in mouse adult testis since at this stage *Catsper1* and *Catsper1au* are expressed from the *Catsper1* bidirectional promoter. To determine the full-length *Catsper1au* sequence, first the 3′-mRNA sequence was determined by 3′-RACE using the oligonucleotides indicated in Table 1. *Catsper1au* was subsequently amplified by RT-PCR using primers flanking the 5′ and 3′ ends. The resulting cDNA was cloned and sequenced to complete the whole nucleotide sequence. An unspliced 1402-bp transcript was obtained (Fig. 4a). This expression was confirmed using a Northern blot assay, where a ~1.4-kb band was detected (Fig. 4b). In addition, only a single band was apparently observed, although the two TSSs were determined by 5′-RACE in the same tissue. Either a single transcript is only produced or the difference of 67 bp between the two transcripts is difficult to distinguish from the Northern blot analysis. However, the size did not correspond with the *in silico*-predicted transcript length. A putative Kozak sequence starts at −699 bp and the poly (A) signal sequence with a single nucleotide change at −1,614 bp relative to the TSS-C. Surprisingly, no significant open reading frames (ORFs) containing a Kozak consensus sequence and longer than 100 base pairs were observed throughout the 1402-bp transcript (Fig. 4c). This analysis might indicate that this transcript is a lncRNA. In fact, the RNAfold server predicted a complex stable secondary structure for this transcript, a major feature of lncRNAs to fold into thermodynamically stable secondary and higher-order structures (Fig. 4c). To ascertain whether this RNA is translated, an *in vitro*-coupled transcription and translation assay was performed. To this aim, the pSdC1 construct, derived by cloning the 1402-bp *Catsper1au* gene under the T7 promoter in the pSG5 vector, was used to prime the reaction using a rabbit reticulocyte system in the presence of biotinylated lysine. The luciferase gene, encoding a 61-kDa protein, was used as a positive control (Fig. 4d). No protein products were detected, suggesting that *Catsper1au* is a lncRNA transcript. To verify whether *Catsper1au* is indeed transcribed, we transfected pSdC1 into adult primary germ cells, and *Catsper1au* was analysed using RT-PCR. As expected, compared with basal expression, *Catsper1au* was overexpressed (Fig. 4e).

### *Catsper1au* is a nuclear lncRNA located in testis germ and somatic cells but not in sperm cells

**Figure 5:**
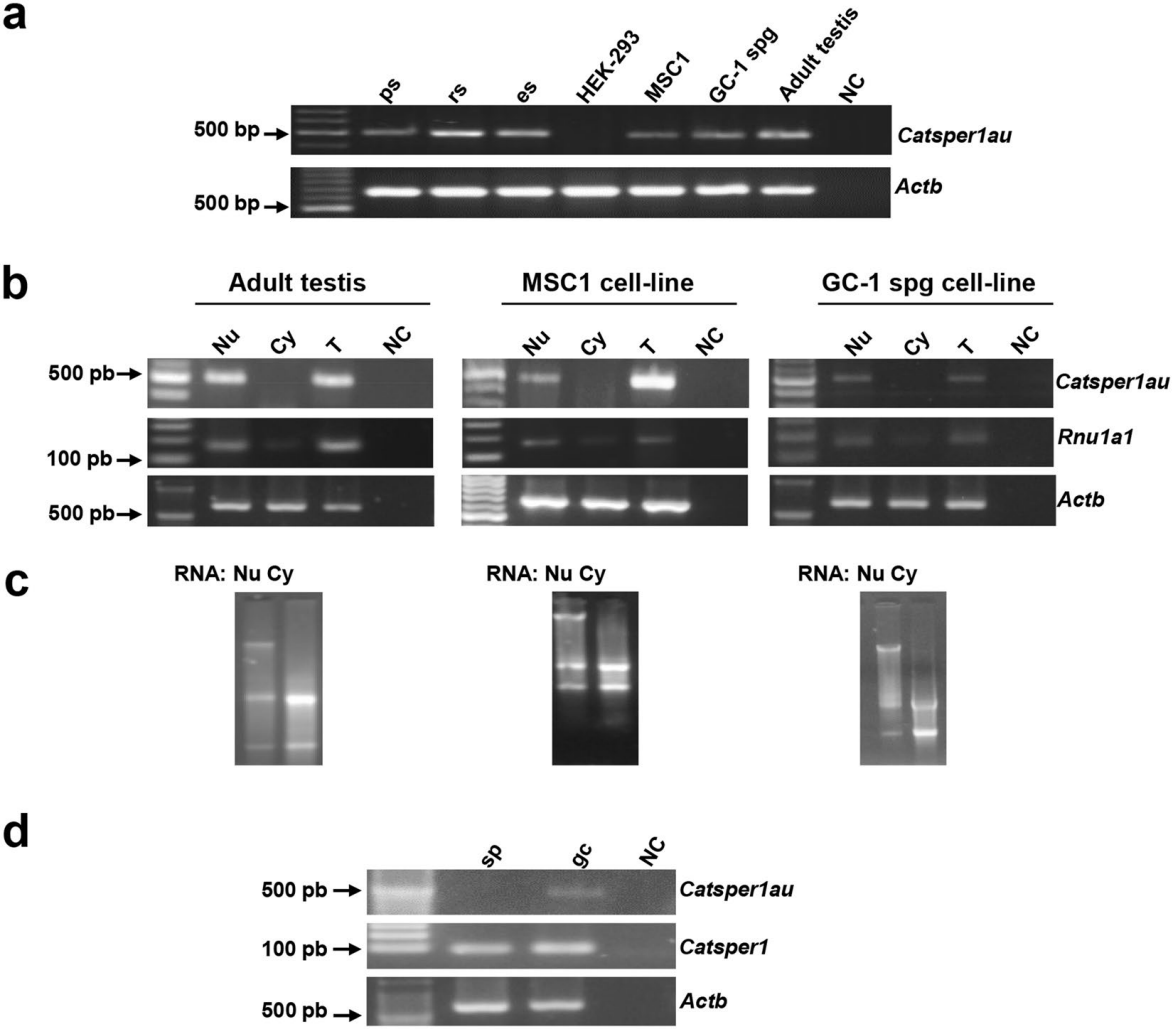
The lncRNA *Catsper1au* displays a nuclear localization and is expressed in germ and somatic cells from testis. (**a**) *Catsper1au* expression in cell lines and testicular germ cells at different stages of spermatogenesis. (**b**) Nuclear and cytoplasmic expression of *Catsper1au* RNA in testis, MSC1, and GC-1 spg cells. *Rnu1a1* was used as a control to verify the quality of fractions. (**c**) Electrophoretic analysis of RNA fractions in a 1.2% agarose gel. (**d**) *Catsper1au* is not expressed in sperm cells. *Actb* was analysed as an internal expression control and to verify the absence of genomic DNA. ps: pachytene spermatocyte; rs: round spermatid; es: elongated spermatid; sp: sperm cell; gc: germ cells.

The subcellular localization of *Catsper1au* was analysed in pachytene spermatocytes, round and elongated spermatids, mouse Sertoli cells (MSC1) and GC-1 spg cells since *Catsper1au* was amplified from these cells using RT-PCR (Fig. 5a). Mouse adult testis tissue, MSC1, and GC-1 spg cells were fractionated into the nucleus and cytoplasm by differential centrifugation, and RNA was subsequently isolated (Fig. 5c). RT-PCR from *Catsper1au* was performed using specific primers. *Catsper1au* was only amplified from both total and nuclear RNA but not from cytoplasmic RNA (Fig. 5b). *Rnu1a1*, a small nuclear RNA (snRNA) component of the spliceosome located in the nucleus^27^, was used to assess the purity of the nuclear and cytoplasmic fractions. Amplification from the nuclear fraction was higher than that from the cytoplasmic fraction. Thus, *Catsper1au* RNA was predominantly detected in the nucleus, which is not surprising since numerous lncRNAs preferentially show nuclear localization^28^. To analyse *Catsper1au* expression in different subpopulations of germ cells, these cells were fractioned into pachytene spermatocytes, round and elongated spermatids using a BSA gradient. *Catsper1au* was specifically amplified by RT-PCR from these fractions (Fig. 5a); however, *Catsper1au* was not detected in differentiated sperm cells obtained from epididymis (Fig. 5d).

**Figure 6:**
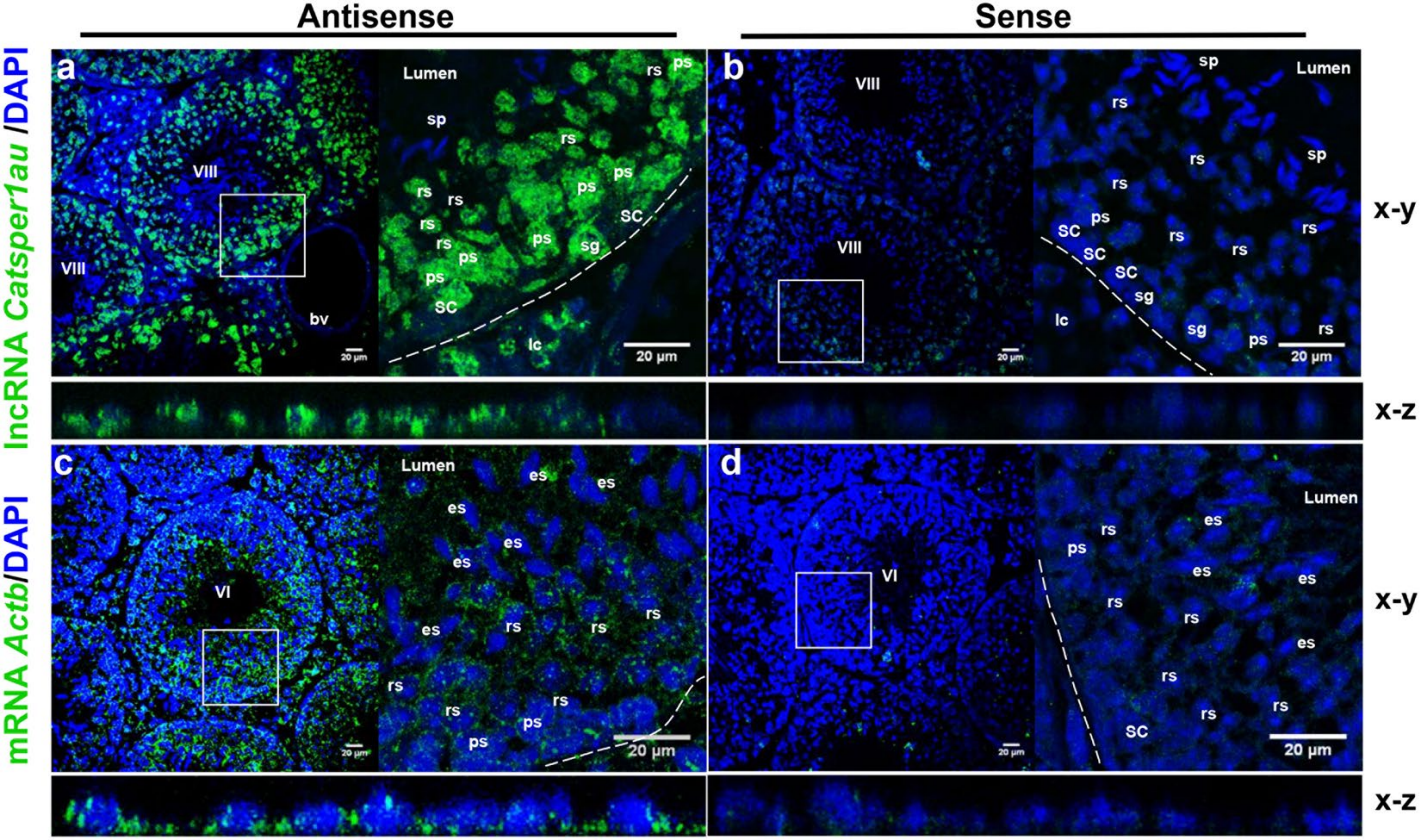
*Catsper1au* is located *in vivo* in the nucleus of spermatogenic and somatic cells from the seminiferous tubule. (**a**) *Catsper1au* RNA was analysed in the mouse testis by FISH using antisense RNA probe derived from *Catsper1au*. (**b**) Sense *Catsper1au* RNA was included as a negative control. Antisense (**c**) and sense (**d**) *Actb* RNA probes were also included as controls. Dashed line, basal border of seminiferous tubule; VI and VIII, stages in the cycle of the seminiferous epithelium; SC: Sertoli cell; sg: spermatogonia cell; ps: pachytene spermatocyte; rs: round spermatid; es: elongated spermatid; sp: sperm cell. Blood vessel: bv; lc: Leydig cell.

The subcellular localization and expression of *Catsper1au* in different germ cell subpopulations were further examined *in vivo* using RNA fluorescence *in situ* hybridization (FISH) on adult mouse testis, using an antisense probe derived from *Catsper1au*. Figure 6a shows that *Catsper1au* RNA localizes at the seminiferous tubules in the nucleus of somatic Leydig and Sertoli cells, spermatogonia cells, pachytene spermatocytes and round spermatids but is absent in the nucleus of sperm cells. A *Catsper1au* sense probe yielded a faint signal (Fig. 6b). In addition, RNA-FISH using an *Actb* antisense probe as a positive control, produced a specific signal both in the nuclei and cytoplasm of all cells present in the seminiferous tubule (Fig. 6c), while the *Actb* sense probe yielded a weak signal (Fig. 6d). Compared with *Actb*, higher concentrations of the riboprobe and anti-digoxigenin antibody (see Materials and Methods) were used to detect lncRNA *Catsper1au* based on its reduced expression observed using qRT-PCR (Fig. 3). The RNA-FISH analysis confirmed the nuclear localization of *Catsper1au*. These findings also corroborated the lack of *Catsper1au* signal in spermatozoa and its expression in pachytene spermatocytes, round and elongated spermatids (Fig. 5). Taken together, the RT-PCR and RNA-FISH results indicated that *Catsper1au* is a lncRNA present in the nuclei of germ and somatic cells of the testis, but not in sperm cells.

## Discussion

In the present study, we demonstrated that the *Catsper1* promoter is bidirectional in GC-1 spg spermatogonial cells. A novel lncRNA (*Catsper1au*) is expressed divergently from the *Catsper1* promoter. *Catsper1au* and *Catsper1* TSSs are separated by 224 bp. Similar to *Catsper*, *Catsper1au* is expressed in adult testis and spermatogenic cells but also in Sertoli cells and liver tissues. No signal was observed in the ovary. In addition, *Catsper1au* is expressed from embryonic to adult stage in the testis, in the 1- to 3-week postnatal heart and in one week to adult stage liver, and unlike *Catsper1*, it is not detected in mature sperm cells. In addition, *Catsper1au*, which encodes a highly structured 1402-bp lncRNA, is located in the nucleus of Sertoli and germ cells from adult testis.

Because of the importance of *Catsper1* in the biology of reproduction, the characterization of its promoter is an interesting study model since it is expressed with high specificity and site-temporality during spermatogenesis. The *Catsper1* promoter bidirectional activity previously reported^20^ was confirmed in the present study in homologous GC-1 spg cells (Fig. 1b). However, a lower activity was detected in the sense direction, consistent with a lack of *Catsper1* expression in spermatogonial cells *in vivo*. In addition, GC-1 spg cells do not express the CREMτ transcription factor, which is involved in gene expression in haploid spermatids during spermiogenesis^25,29^, a stage where *Catsper1* is expressed^15^. Nevertheless, it has been reported that the activity of a bidirectional promoter may be regulated by orientation-dependent *cis*-control elements or trans-acting factors. These mechanisms could positively regulate the expression of *Catsper1au* in GC-1 spg cells^24^.

Another feature of bidirectional promoters is that the TSSs of divergent genes are separated by less than 1 kb^30^. The intergenic region between *Catsper1* and *Catsper1au* divergent genes is consistent with the characteristics of bidirectional promoters^30^ since the two TSSs identified for *Catsper1au* are −224 and −291 bp upstream of the *Catsper1* TSS (Fig. 2b). The majority of divergent gene pairs are co-regulated, have related functions, function in a common pathway and show tissue-specific expression^24^ similar to *Col4a1* and *Col4a2*, *HAND2* and *HAND2-AS1* ^31,32^. Although *Catsper1au* and *Catsper1* are expressed in adult testis, suggesting that both genes are co-regulated through their shared proximal promoter, *Catsper1au* transcripts is unidirectionally expressed in adult liver. Therefore, these genes do not exhibit the same tissue-specific expression pattern (Fig. 3a). The genes regulated by a bidirectional promoter are not always co-expressed or co-regulated and may even be functionally unrelated, such as *SERPINI1* and *PDCD10* ^24^. Interestingly, similar to *Catsper1*, *Catsper1au* is only expressed in male gonads but not in female gonads, potentially suggesting a specific role in the development of the male reproductive system (Fig. 3b), associated with the expression of *Catsper1au* in the embryonic stage (11.5 d.p.c.). Mammalian sex differentiation relies on the expression of the *Sry* gene, which in mouse begins at 10.5 d.p.c and peaks at 11.5 d.p.c^33^. At this stage, the participation of *Sry* and a set of male-specific genes in testis development might involve *Catsper1au* (Fig. 3c). However, *Catsper1au* was not expressed in the adult female liver (Fig. 3b). In this respect, male and female liver display sex-dependent gene-expression primarily regulated by pituitary GH, whose regulation is driven by oestrogen and testosterone sex hormones^34^. In this sense, *SULT2A*, a gene that participates in hydroxysteroid sulphate conjugation, is repressed in the adult male liver, while the transcript is only expressed in the adult female liver^35^. Thus, the *Catsper1au* expression in the male liver could be hormonally regulated. Additionally, *Catsper1au* is expressed in newborn to four-week testis and continues until the adult stage, while *Catsper1* expression is restricted to adult testis. Accordingly, the expression throughout mouse life also suggests that *Catsper1au* might have a function in testis development and male germ cell differentiation. The first cycle of spermatogenesis in the mouse starts after birth, and the time points at which spermatogenic cell types appear are well defined^36^. Indeed, *Catsper1au* expression is higher in adolescent 4-week mice, where the spermatids reach the elongation phase, suggesting an important role at this stage.

Surprisingly, *Catsper1au* is expressed in 1-to 3-week-old but not in adult mouse hearts. The transition of cardiomyocytes to differentiated cells that are unable to proliferate occurs in the first two weeks post birth in the mouse heart^37^. At this time, the expression of genes, such as *FRNK*, has been implicated in this process, peaking at days 5 to 7 post birth and decreasing in the adult stage^38^, prompting the question of whether *Catsper1au* plays a role at this stage. *Catsper1au* expression in the liver was initiated from the first week and continued to the adult stage. Liver gene expression at one week after birth matches with the activation of genes involved in lipid and fatty acid metabolism^39^ and the genes that promote liver growth, such as *Mest*, *Peg3*, and *Igf2* ^34^. *Catsper1* expression is initiated in the third week in Balb/C mice, unlike in CD-1 mice, where this expression is detected in four-week-old mice^40^. Diverse genes, with relevant functions in adult testis, show a differential expression pattern throughout mouse life, such as *Dmrt1*, *Mtl5*, *NYD-SP5* ^41–43^. Some of these genes are also expressed in other organs. For example, *Mtl5n* is also expressed in the foetal heart and ovary and in the adult heart^42^. Although *Catsper1au* has an expression profile different from that reported for *Catsper1* ^7^, these genes may be functionally related during their co-expression in adult testis.

The *in silico* analysis and the lack of translation products indicate that *Catsper1au* gene encode a lncRNA. These novel RNA molecules are more than 200 nt in length, primarily unspliced and polyadenylated, do not encode proteins and the primary sequence shows low conservation across evolution, unlike protein-coding genes^2,44,45^. Indeed, *Catsper1au* is unspliced and polyadenylated with a unique variation in a nucleotide, as reported for *Mrhl* lncRNA^46^, and this sequence was not detected in the human genome database (data not shown). Recently, novel TSSs in human lncRNAs genes, upstream of the previously annotated sites, have been identified using RACE-Seq. However, the expected transcripts have not been experimentally identified^47^. Thus, two *Catsper1au* isoforms could be expressed from the two TSSs identified using 5′-RACE. However, only one transcript was detected using Northern blotting. However, lncRNAs are expressed at much lower levels than protein-coding genes and exhibit a tissue/cell or developmental stage specific expression profile^2,45,48^. Indeed, the expression level of *Catsper1au* is less abundant than *Catsper1* (Fig. 1e), which contrasts with the antisense *in vitro* bidirectional promoter activity (Fig. 1b). In the testis, it is likely that lncRNAs play an essential function in testis development and spermatogenesis^2^. For example, *Tsx* (Testes-specific X-linked), a lncRNA expressed in meiotic germ cells and brain tissue, is relevant for the development of germ cells because Tsx-null mice show smaller testis as a result of pachytene germ cell apoptosis^49^. Depending on the function, most of the genes expressed during spermatogenesis display exclusive expression profiles at the different stages of spermatogenic cells^44^. In this regard, *Catsper1au* is expressed both in spermatogonia cells, GC-1 spg cells, pachytene spermatocytes, round and elongated spermatids and in somatic Leydig and Sertoli cells (Figs 5 and 6), which may suggest a role for lncRNA-*Catsper1au* during spermatogenesis.

Bidirectional promoters in the testis may or may not co-regulate a protein-coding gene and a lncRNA. For example, a bidirectional promoter regulates the protein-coding gene *Piwil1*, which is important in meiosis and is exclusively expressed in spermatocytes and spermatid cells, and the lncRNA (*AK016105*). Both molecules show similar expression profiles in adult testis. An opposite expression profile was observed for *Zfp148*, which participates in the development of germ cells, and the lncRNA (*AK160141*). The expression of this lncRNA is lower than that of *Zfp148* in newborn testis^2,50,51^. A bidirectional promoter that regulates the expression of a new lncRNA of unknown function named *lncRNA-Tcam1* and *Smarcd2* in testis has recently been reported. While *lncRNA-Tcam1* is specifically expressed only in the testis in the nucleus of germ cells, *Smarcd2* is expressed in all tissues. Therefore, this promoter is bidirectional in testicular germ cells and unidirectional in other tissues^52^, as is the case of the *Catsper1* bidirectional promoter reported here.

Although most of the lncRNAs are located in the nucleus, some lncRNAs are located in the cytoplasm or both compartments^53^. The subcellular location of lncRNAs may be related to its function; for example, in the cytoplasm, lncRNA generally functions at the post-transcriptional level, such as *BACE1-AS*. In the nucleus, these molecules can act as guides for chromatin-modifying complexes at the epigenetic level. For example, *XIST* participates in the transcriptional regulation of proteins, such as *Air* and *HOTAIR* and regulates the alternative splicing of pre-mRNA, such as *lncRNA-p21* ^54–56^. *Catsper1au* plays an important role in gene regulation considering that this gene is expressed in the nucleus of germ and somatic cells in the testis, and this lncRNA was not detected in transcriptionally inactive mature sperm cells^57^. lncRNAs derived from bidirectional promoters can activate or repress the transcription of their neighbouring protein-coding genes *in cis*, such as *Six3OS*, or distant genes in *trans*, such as *Vax2OS1*, through epigenetic mechanisms^54^. However, additional studies are needed to characterize the *Catsper1* bidirectional promoter and examine its role in the expression of both genes. It is also necessary to knock out *Catsper1au in vitro* and *in vivo* to assign a biological function to this novel lncRNA. However, these data strongly suggest that *Catsper1au* participates in the regulation of gene expression during spermatogenesis.

## Materials and Methods

### Source of tissues

ICR (CD-1) mice were obtained from the Institutional Animal Care and Use Committee (IACUC) of the Center for Research and Advanced Studies (IACUC-CINVESTAV). Animal handling and all experimental protocols were fully accredited and performed in accordance with the Ethical Guidelines and Procedures from the IACUC-CINVESTAV, protocol number 0113-14. IACUC-CINVESTAV is the regulatory office for the approval of research protocols involving the use of laboratory animals and fulfils the Mexican Official Norm (NOM-062-ZOO-1999) “Technical specifications for the Care and Use of Laboratory Animals”.

### Transient transfection and luciferase assays

Mouse spermatogonial cells GC-1 spg (ATCC® CRL2053™) were cultured in DMEM (Sigma) supplemented with 10% (v/v) foetal bovine serum at 37 °C under a humidified atmosphere and 5% CO_2_. Cells were seeded in 24-well culture (2. 5 × 10^5^ cells/well) at a density such that the cells reached 70–80% confluency at the time of transfection. GC-1 spg cells were transiently transfected using TurboFect™ *in vitro* (Fermentas) reagent. The transfection mixture was prepared with 1 µg of each pGL3-Basic derived constructs containing *Catsper1* promoter in either direction upstream the luciferase reporter gene. pRL-CMV (0.2 ng) was used as a control to evaluate the transfection efficiency and normalize the data. Luciferase assay was performed using a Dual Luciferase system (Dual- Luciferase Reporter Assay; Promega) after 48 h of incubation.

### *In silico* analysis of the 10.4-kb *Catsper1* divergent region

The genomic sequence of the 10.4-kb region was analysed *in silico* to predict splicing sites and putative exons using GeneMark.hmm, NetGene2 and FGENESH 1.1 bioinformatics Web Servers. The TSS and poly(A) sites were analysed using Neural Network Promoter Prediction and POLYAH SOFTBERRY, respectively. The presence of CpG islands was analysed using CpGFinder. EST search was performed with NCBI BLAST service. RepeatMasker programme was used to identify DNA repeat sequences. RNA secondary structure was predicted with RNAfold. Transcription factor binding sites (TFB) were predicted with TRANSFAC database.

### 5′/3′-RACE

*Catsper1au* 5′ and 3′ ends were determined using the primers shown in Table 1 and the Marathon cDNA Amplification kit (Clontech) according to the manufacturer’s instructions. Reverse primers for 5′-RACE were designed downstream of the two putative TSSs predicted *in silico* for *Catsper1au*. For the first reaction of a nested PCR; AS322, AS219, and P1200R primers were used, each in combination with the forward primer adapter AP1, to generate the products AS322-AP1, AS219-AP1 and P1200R-AP1. In the second reaction, a 1:50 dilution of the cDNA products of the first reaction was used as template together with the reverse d3R primer and the forward internal adapter AP2. For the 3′-RACE, forward primers were designed based on the sequence obtained by 5′-RACE. In the first PCR, we used the 3′RACE-Forw1 primer, and the reverse adapter AP1. In the second reaction, 3′RACE-Forw2 and the reverse internal adapter AP2 were used. The PCR fragments generated in the 5′/3′-RACE were analysed using 5% acrylamide gel electrophoresis, cloned into pJET 1.2/blunt (ThermoFisher Scientific) and 20 clones were sequenced using the BigDye terminator sequencing kit Version 3.1 (Applied Biosystems). *Catsper1au* was amplified by RT-PCR using primers NGF225 and NG-Reverse that flanked the 5′ and 3′ sequences to generate and determine the full-length sequence; cloned into pJET 1.2/blunt and sequenced. The whole sequence of *Catsper1au* was annotated in the NCBI Database, accession number KX825862 and *Catsper1au* (*Catsper1* antisense upstream transcript) was named according to The Jackson Laboratory, MGI Nomenclature Committee.

### RT-PCR

Germ cells from adult (12-week-old) mouse testis were fractioned on a 2–4% BSA linear gradient into pachytene spermatocyte, round and elongated spermatids according to a previous report^58^. Sperm cells were obtained from epididymis as previously described^59^. Total RNA was isolated from mouse male and female tissues, germ cells, sperm cells; and from MSC1 and GC-1 spg cell lines by the phenol extraction method with TRIzol (Invitrogen^™^) and treated with RQ1 RNAse-free DNAse (Promega). RNA from mouse thymus and 11.5-day embryo was purchased from Clontech. The sex of the embryo was verified as shown in Supplementary Fig. 1. The cDNA was obtained from 2 µg of total RNA using a transcription High capacity RNA reverse transcription kit (Applied Biosystems). *Actb* was used as an internal expression control and to verify the absence of genomic DNA in the cDNA samples. Primers Act(F) and Act(R) generate a 660-bp *Actb* product and a band of 880-bp in the case of genomic DNA contamination. A 93-bp *Catsper1* product was PCR amplified from testis cDNA (30 cycles and using 1 µl from the cDNA stock solution diluted 1:20) with primers Cat1 (F) and Cat1 (R). These primers were designed between exons 2 and 3 of *Catsper1*. Additionally, *Catsper1* was amplified using 35 cycles and 1 µl of cDNA to verify the absence of genomic DNA in all the samples. A 1.2-kb product indicates genomic contamination. Primers NGF225 (F)-AS306 (R), NGF292 (F)-AS306 (R) and NGF225 (F)-NG-Reverse (R) were used to obtain the 496 bp, 429 bp and 1402 bp *Catsper1au* products, respectively. *Catsper1au* was PCR amplified from testis cDNA (40 cycles and using 1 µl from the cDNA stock solution). PCR products from *Catsper1* and *Catsper1au* were sequenced to confirm the identity of the fragments.

### Quantitative real-time PCR

The relative expression of *Catsper1au* RNA and *Catsper1* was measured by real-time quantitative PCR (LightCycler® 480 System, Roche) using SYBR® Premix assay (Roche) with the primers NGFORW225RT (F)-REVD3 (R) and Cat1(F)-Cat1(R). The primers ActBForw (F) and ActBRev (R) were used for normalization and were designed from the *Actb* coding region (Table 1). The relative expression of mRNA was calculated using the ΔΔCt method^60^. Products were initially confirmed by sequencing. A final melting curve was also generated to ensure a single PCR product was produced in the reaction. Three independent experiments with three technical replicates were performed.

### Northern blot analysis

Total RNA (10 µg) from adult testis or heart was loaded onto agarose gels containing guanidine thiocyanate (5 mM), run for 3 h and subsequently alkaline capillary transferred to a positively charged nylon membrane for 2.5 h. The blots were hybridized for 16 h at 62 °C with digoxigenin-UTP-labelled riboprobe for *Catsper1au* synthetized according to the manufacturer’s instructions in the DIG RNA labeling Kit (Sp6/T7) (Roche). A non-radioactive chromogenic method was used for probe detection. Briefly, after washing and blocking, the membrane was incubated with Anti-Digoxigenin-AP 1:2500 (Roche, Cat. 11093274910) and incubated for 16 h with BCIP®/NBT Liquid Substrate (Sigma, Cat. B1911).

### *In vitro* coupled transcription-translation

The *in vitro* coupled transcription-translation reaction was performed with the TNT® Coupled Reticulocyte Lysate System. *Catsper1au* was amplified from adult testis cDNA using primers ENGFORW225 and BRACE3REV and cloned downstream of the T7 promoter into the pSG5 vector. Briefly, 1 µg of circular plasmid was added to TNT® Lysate and incubated in a 50-µl reaction for 90 min at 30 °C. To label the protein, Biotinylated lysine (Transcend™ Biotinylated tRNA) was included. The products were resolved on a 15% SDS-PAGE. A non-radioactive chemiluminescent system was used for protein detection. A luciferase plasmid encoding a 61-kDa protein and the pSG5 empty vector were used as positive and negative controls, respectively.

### Overexpression of *Catsper1au* in primary culture of germ cells

Spermatogenic cells were obtained from two-month postpartum mouse. Testes maintained in DMEM were minced into small pieces, digested with 4-mg/ml type IV collagenase (Sigma-Aldrich) at room temperature for 30 min and washed with DMEM to obtain seminiferous tubules. Germ cells were obtained by pipetting the seminiferous tubules. Cell clots were removed and cells selected by filtration in 80 μm mesh. Two million cells were seeded onto 6-well plates with DMEM/F12 supplemented with nonessential amino acids, 15 mM HEPES, 0.12% sodium bicarbonate, 100 IU/ml penicillin, 100 μg/ml streptomycin, 30 mg/ml pyruvic acid, 5 µg/ml insulin, 2 mM L-glutamine, 5 µM vitamin E, 550 ng/ml FSH, 0.1 µM testosterone, and 10% FBS. The cells were subsequently transiently transfected with pSG5 plasmid (which expresses *Catsper1au* transcript) and Lipofectamine 3000 (Invitrogen), harvested 48 h later and maintained in TRIzol for RT-PCR analysis.

### Subcellular fractionation

Mouse adult testis tissue, MSC1, and GC-1 spg cells were fractionated into nucleus and cytoplasm by differential centrifugation, and total RNA was isolated. Briefly, cells were suspended in 1 × PBS and centrifuged at 300 × g. The pellet was resuspended in ice-cold cell lysis buffer (50 mM Tris-HCl at pH 7.4, 5 mM MgCl_2_, 100 mM NaCl, 1 mM dithiothreitol (DTT), 0.5% (v/v) Nonidet P-40, 25 U/µl RNasin® (Promega), and centrifuged 10 min at 17,000 × g and 4 °C. The cytoplasmic RNA was isolated from the supernatant by phenol extraction. The nuclear pellet was resuspended in lysis buffer and incubated for 15 min on ice, and sucrose was subsequently added to 2.3 M and centrifuged at 20,000 × g 4 °C for 30 min. Nuclear RNA was isolated using TRIzol and treated with RQ1 RNAse-free DNAse.

### RNA-FISH

Testis from adult mice was cut into 8 μm sections using a cryostat (Leica MC1510) and mounted at −4 °C on gelatin- coated slides. Slides were fixed with 4% p-formaldehyde and treated with 10-μg/ml proteinase K. The sections were acetylated and permeabilized with 0.4% Triton X-100 in PBS. Samples were prehybridized for 30 min in prehybridization buffer (50% formamide, 4x SSC, 20% dextran sulphate sodium) and incubated with digoxigenin-UTP-labelled riboprobe for 12 h at 60 °C (800 and 200 ng/ml for *Catsper1au* and *Actb* probes, respectively). The sections were first washed with 50% formamide, 4x SSC and 10% SDS and subsequently with 50% formamide and 2x SSC. After blocking with 0.1% Tween and 3% BSA in PBS, the sections were incubated for 1 h with anti-digoxigenin-fluorescein Fab fragments diluted 1:100 and 1:200 for *Catsper1au* and *Actb* probes, respectively (Roche, Cat. 11207471910). Slides were mounted with Vectashield with DAPI (Vector Burlingame, CA), and the images were captured using a confocal microscope (Leica Sp8). The sense and antisense riboprobes for *Catsper1au* and *Actb* were synthesized according to the manufacturer’s instructions in the DIG RNA Labeling Kit (Sp6/T7) (Roche). Briefly, *Catsper1au* and *Actb* were amplified from adult testis cDNA using primers HNGF225 (F)-ECOAS306 (R) and ActH3 (F)- ActECO (R) respectively and cloned downstream of the SP6 promoter and upstream of the T7 promoter into the pSPT 18 vector.

### Statistics analysis

Statistical analysis was performed with a one-way ANOVA test using GraphPad Prism 7.01 following Dunnett’s multiple comparisons test for luciferase data analysis. The level of significance was *P < 0.05, **P < 0.01, ***P ≤ 0.001. Data were expressed as the mean ± standard deviation (SD), n = 3. qRT-PCR data were also analysed using one-way ANOVA, followed by Bonferroni post hoc test. Differences among groups are indicated as letters, where a > b (Fig. 3d). Statistical significance was ***P ≤ 0.001 using paired 2-tailed t-test (Fig. 3e). Data were expressed as the means ± standard error (S.E); n = 3.

## Electronic supplementary material

Supplementary PDF File
