# Supplementary material for: Catsper1 promoter is bidirectional and regulates the expression of a novel lncRNA
Source: Sci Rep. 2017 Oct 17;7:13351. doi: 10.1038/s41598-017-13867-2 (PMC5645346; doi:10.1038/s41598-017-13867-2)
Supplement: Supplementary file 1 — Supplementary PDF File [file 41598_2017_13867_MOESM1_ESM.pdf]

***Catsper1* promoter is bidirectional and regulates the expression of a novel lncRNA.**

Salma E. Jiménez-Badillo, Norma Oviedo, Christian Hernández-Guzmán, Lorenza González-Mariscal, and Javier Hernández-Sánchez*

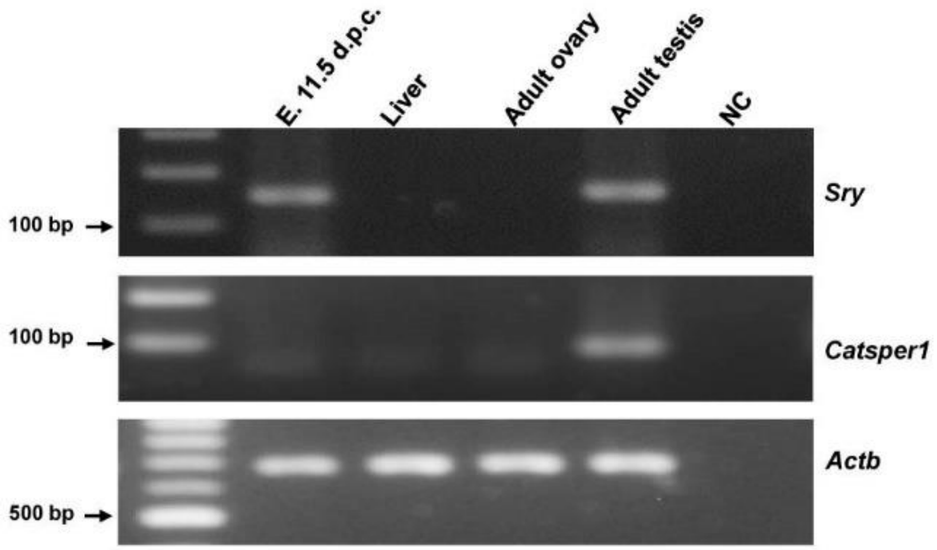

Name	**Sequence (5'→3')**
SRYForw (F)	ATTTATGGTGTGGTCCCGTG
SRYRev (R)	ATCTTCAATCTCTGTGCCTCC

Supplementary Fig. 1. *Sry* is amplified from embryo 11.5 d.p.c. and adult testis cDNA. A 156 bp *Sry* product was PCR amplified from embryo 11.5 d.p.c. and adult testis cDNA using SRYForw (F) and SRYRev (R) primers. RNA from adult liver and ovary was included as a negative control. *Actb* was analysed as an internal expression control to verify the absence of genomic DNA. The negative control (NC) is shown in the last lane. E: embryo; d.p.c: days post coitum.

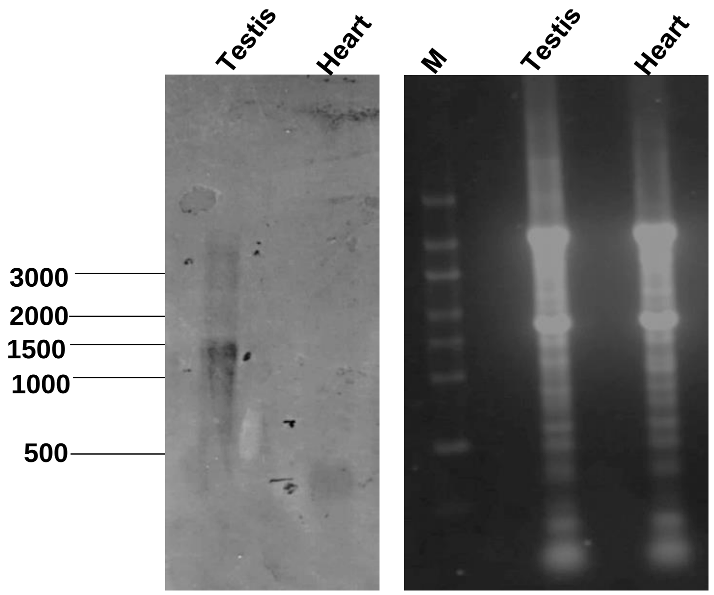

Supplementary Fig. 2. Northern blot analysis of *Catsper1au* expression in mouse testis and heart. RNA from adult testis and heart was resolved on 1.2% agarose gels, transferred onto nylon membranes and *Catsper1au* was detected as indicated in the Materials and Methods section. A ~1.4-kb band corresponding to *Catsper1au* is detected in mouse testis (left panel). Ethidium bromide staining of ribosomal RNA (right panel). M, RNA ladder (high range).

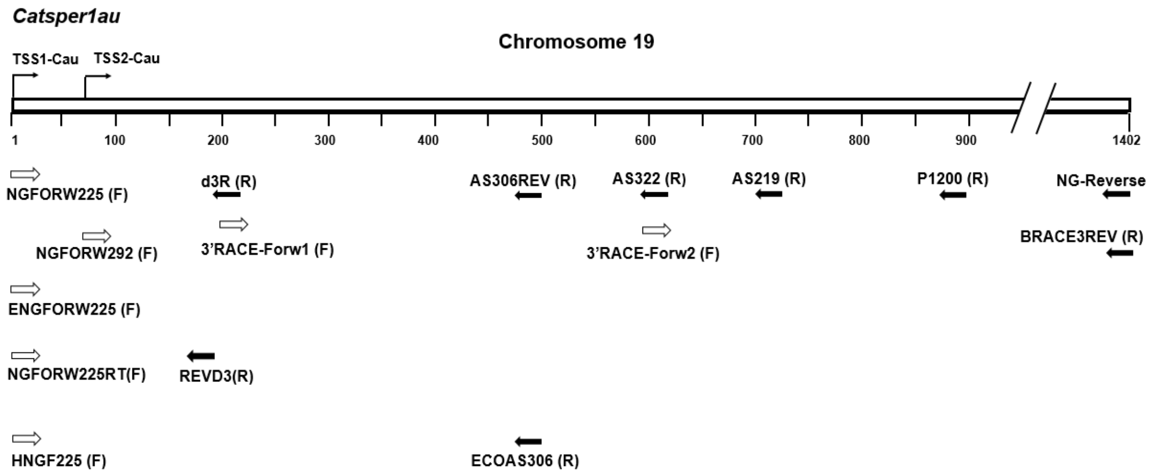

Supplementary Fig. 3. Primer location in *Catsper1au* locus. *Catsper1au* is located in chromosome 19, (location: complement (5334032-5335433)). Bent arrows represent *Catsper1au* TSSs. Arrows represent Forward and Reverse primers used in RACE5'/3', RT-PCR, molecular cloning of *Catsper1au,* qPCR, Northern blot and RNA-FISH.
